# Oxidized Oligosaccharides Stabilize Rehydrated Sea Cucumbers against High-Temperature Impact

**DOI:** 10.3390/ijms21155204

**Published:** 2020-07-23

**Authors:** Jingyi Liu, Yanan Xu, Tianhang Xia, Changhu Xue, Li Liu, Pengtao Chang, Dongfeng Wang, Xun Sun

**Affiliations:** College of Food Science and Engineering, Ocean University of China, Qingdao 266003, China; Jingyi_liu30@163.com (J.L.); xuyanan152@163.com (Y.X.); 18861807285@163.com (T.X.); xuech@ouc.edu.cn (C.X.); 17852152951@163.com (L.L.); ChangPengT@163.com (P.C.); wangdf@ouc.edu.cn (D.W.)

**Keywords:** oxidized oligosaccharides, sea cucumber, hydrogel, deterioration, diffusion, crosslinking

## Abstract

Small-molecule crosslinkers could diffuse into and stabilize protein hydrogels without damaging their appearance, but they are absent from the food industry due to the high safety and efficacy requirements for foods. Oxidized oligosaccharides are non-toxic small polyaldehydes previously found capable of crosslinking proteins by premixing. In this study, we managed to diffuse various oxidized oligosaccharides into the protein wall of rehydrated sea cucumbers, and the texture profile analysis, total soluble material assay and SEM (scanning electron microscope) images all suggested the treated sea cucumbers acquired significantly enhanced stability against high-temperature-promoted deterioration. The stabilization was positively correlated with the aldehyde content of oxidized oligosaccharides but negatively correlated with molecular size. The mechanism of stabilization was found to include both covalent and hydrogen bond crosslinking. These results have demonstrated that oxidized oligosaccharides could enter food protein hydrogel by free diffusion and stabilize the 3D network effectively and thereby has great potential in food-related industry.

## 1. Introduction

It is known that crosslinking may introduce favorable properties to the appearance [1], stability [2,3], texture [4] or even taste [5] of protein hydrogels. In the food industry, enzymes, introduced by premixing, have successfully crosslinked many highly dispersed protein systems (e.g., sausages and protein suspensions), yet they fail to work as efficiently in heterogeneous systems (e.g., shrimps and whole sea cucumbers) because these macromolecules [6] are too sterically hindered to diffuse into the deeper part of protein hydrogel. In contrast, small-molecule crosslinkers could easily diffuse into protein hydrogels from solution and thereby are more competent in crosslinking heterogeneous systems.

The development of new small-molecule crosslinker in the food field progresses slowly, largely because of the high requirements of safety and efficacy for food crosslinkers. Mono- and oligosaccharides are small enough to diffuse into protein hydrogel and have been found capable to crosslink proteins through Maillard reaction [7]. This complex sequence involves multiple reversible dehydration reactions as intermediate steps [8]. However, protein hydrogel is a highly water-rich system which severely suppresses these dehydrations, and therefore saccharide crosslinking is often only successful on hydrogel surface where water releases faster. Genipin is a natural small molecule allowed for human use, and could well crosslink protein hydrogels [9,10], yet it dyes proteins to blue color, which in foods are often considered unhealthy by the general consumers. Many other small molecules could well crosslink proteins to form new materials [2], yet their success could not be repeated in food context due to the strict safety requirement for foods. For example, glutaraldehyde has been widely used in the synthesis of protein hydrogel-based new materials, yet they are prohibited for food use in European Union and the United States due to its high toxicity [11]. So far, a safe and effective small-molecule crosslinker for protein hydrogel is still absent in the food industry.

We propose this absence could potentially be filled with oxidized oligosaccharides. Oxidized oligosaccharides are a type of polyaldehyde typically generated by oxidizing sugars with NaIO_4_. These organic compounds bear multiple aldehyde groups in each molecule which can crosslink protein hydrogel by imination with the amine groups on different peptide chains and provide extra stability against impacts. The molecular size of oxidized oligosaccharides depends on the size of their precursors, so mono- and oligosaccharide-derived ones are reasonably small and could access the deep part of protein hydrogel by free diffusion from a heterogeneous phase. However, to the best of our knowledge, so far this type of compound has only been used in highly dispersed systems by premixing, such as solutions of chitosan [12], corn starch [13], atelocollagen [14] and keratin [15], but no study on a heterogeneous system has been reported. Furthermore, compared with normal polyaldehydes, oxidized oligosaccharides bear abundant hydroxyl groups which provide higher water solubility and biocompatibility [15,16]. For example, oxidized sucrose has been found non-toxic according to in vitro cell studies [17,18].

Rehydrated sea cucumber is a good subject to test oxidized oligosaccharides on their diffusing and crosslinking ability in a food matrix. Sea cucumber (Apostichopus Japonicus) is a high-value seafood favored in northeast Asian countries (especially China) [19]. Rehydrated sea cucumbers from dried or salted ones are more favored than the fresh since they exhibit better gumminess and flavor and are thereby considered an ideal material for deeper and value-added products, such as ready-to-eat stewed sea cucumber and cucumber–millet porridge. The major edible part of sea cucumbers is their body walls which is mainly collagen hydrogel [20] that deteriorates severely at high-temperature sterilization (121 °C, 10 min). Heat-promoted deterioration typically softens sea cucumber body wall and increases soluble materials, which cause serious damage to rehydrated sea cucumbers, so it is currently one of the biggest problems limiting the development of ready-to-eat products from rehydrated sea cucumbers. Our group has previously suggested that deterioration is caused by heat-promoted breakage of chemical bonds in sea cucumber collagen [21], which could be avoided by extra crosslinking. The commercial value of sea cucumber relies heavily on its unit size and integrity, so crosslinking agents have to be introduced without damaging the appearance of sea cucumbers, preferably by free diffusion.

Our earlier effort using lignin-rich herb extracts [22] and Ca^2+^ bridge [23] to stabilize rehydrated sea cucumbers only received marginal success; other more recent attempts such as crosslinking with citric acid, mono- and oligosaccharides and transglutaminase all failed due to the aforementioned reasons. On the other hand, genipin and glutaraldehyde greatly enhance sea cucumbers’ heat stability, yet the former unsurprisingly dyed sea cucumbers to dark blue color and the latter is legal for food use. Nonetheless, these results showed dialdehydes (genipin is the precursor of a dialdehyde) are capable to crosslink rehydrated sea cucumbers. Oxidized oligosaccharides bear more aldehyde groups in each molecule for crosslinking, so we propose they should also be able to stabilize rehydrated sea cucumber.

The present study was designed to test the feasibility of using oxidized oligosaccharides to stabilize rehydrated sea cucumbers against high-temperature treatment. Rehydrated sea cucumbers were soaked in the solutions of various linear and cyclic oxidized oligosaccharides with different aldehyde contents and then heated at 121 °C for 10 min. The resulting high-temperature-treated (HTT) rehydrated sea cucumbers were characterized with texture profile, degree of imination and total soluble material. The crosslinking mechanism, possible affecting factors and the mechanism of deterioration are discussed.

## 2. Results and Discussion

### 2.1. Preparation and Characterization of Oxidized Oligosaccharides

**Figure 1:**
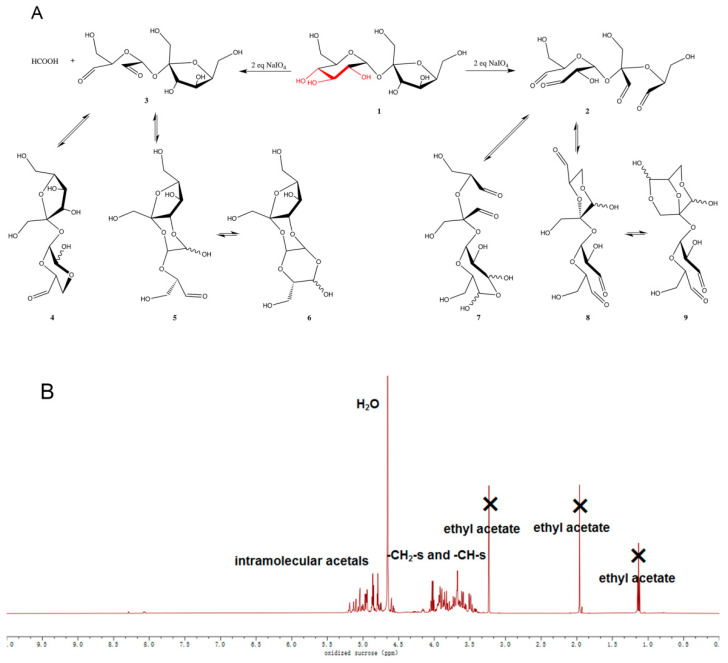
(**A**) Illustrative examples of oxidation of sucrose with NaIO_4_ and subsequent isomerization. The routes selected in this figure are a only a subset of the possible isomerization pathways. The curvy bond represents undetermined configuration. (**B**) ^1^H-NMR spectra of oxidized sucrose.

Oxidized oligosaccharides were synthesized by treating aqueous oligosaccharide solutions with 1.1 equivalence of sodium periodate for each sugar ring and determined the aldehyde content by hydroxylamine chloride titration [24]. As listed in Table 1, the aldehyde contents of the oxidized oligosaccharides were all lower than the theoretical values. This difference is attributed to insufficient oxidation. This reaction oxidatively cleaves vicinal dihydroxide moieties in sugar rings, and optimally, each sugar ring is expected to afford two aldehyde groups at the consumption of one equivalence of sodium periodate. However, in the case of sucrose, raffinose and stachyose, where contiguous triol moieties (Figure 1A, red) exist, a side reaction of double oxidation to that moiety may happen, which consumes two equivalences of NaIO_4_ but only generate two aldehydes. Taking sucrose as an example (Figure 1A, 1), the normal oxidation (Figure 1A, 1→2) yielded four aldehydes upon the consumption of two equivalences of NaIO_4_. Whereas double oxidation to the contiguous triol consumed the same amount of NaIO_4_, it only generated two aldehyde groups from the glucopyrnoside, leaving the fructose unit intact (Figure 1A, 1→3) and thereby lowering the total aldehyde content. This also explains the significantly lower aldehyde content of raffinose and stachyose than the rest sugars, because they bear more contiguous triols in each molecule.

Detailed characterization and structural elucidation of the oxidized oligosaccharides were hampered by their complex isomerizations. Oxidized oligosaccharide belongs to the family of polyol-polyaldehyde and is prone to cyclize by intramolecular acetalization, which results in uncertainties in their structural elucidation. As shown in Figure 1A, taking the oxidation of sucrose as an example, both 2 and 3 bear multiple aldehyde and hydroxyl groups at different positions and are all (at least in theory) susceptible to the reaction, so numerous combinations of cyclizations are possible in the system (e.g., 3 →4 or 5 and 2→7 or 8) and each run of acetalization could yield two different stereoisomers (R/S configurations at the acetal center, or anomers). Moreover, the resulting hemiacetals may either retain its structure or undergo further cyclization to form more complex structures (e.g., 5→6 and 8→9). Taking oxidized sucrose as an example, according to ^1^H NMR (Figure 1B), hardly any free aldehyde group existed in the solution. Instead, the aldehydes were in acetal/hemiacetal form, which are shown in 5.0–6.0 ppm. This result was in agreement with the discussion above. A similar trend was also acquired by Mi et al. [15].

As a result, the oxidation reaction ultimately yielded an inseparable mixture of constitutional isomers and stereoisomers, which could not be analyzed by tradition structural elucidation methods, e.g., NMR. Since full structural elucidation is not the major goal of this research, we decided to only characterize the resulting mixture by the total amount of aldehydes.

### 2.2. Texture Profile Analysis

Preliminary experiments showed that 0.4% to 0.6% (*w*/*v*) oxidized oligosaccharides solution could provide decent stabilization to rehydrated sea cucumbers, so in this study rehydrated sea cucumbers were all heated in 0.5% oxidized oligosaccharide solutions for crosslinking. The crosslinked sea cucumbers were then heated in a sterilizer at 121 °C for 10 min, which according to our previous work [21], could result in severe deterioration to normal rehydrated sea cucumbers.

The deterioration of rehydrated sea cucumber is featured with weakening of mechanical strength and increase of stickiness. Thus, in order to evaluate the stabilizing effect of oxidized oligosaccharides on rehydrated sea cucumbers, the texture profiles of the HTT sea cucumbers were characterized. As shown in Table 2, the hardness, chewiness, gumminess and springiness of the oxidized oligosaccharide crosslinked sea cucumbers were all superior to the control group, which suggested that oxidized oligosaccharides managed to enter the sea cucumber body walls by free diffusion and then enhanced the heat stability of rehydrated sea cucumbers. In particular, normally oxidized sucrose (Table 2, entry 3) showed the best overall performance. In linear oligosaccharide examples (Table 2, entry 3,4 and 5), the mechanical strength was positively correlated with aldehyde content. In order to double-check this trend, we deliberately treated sea cucumbers with partially oxidized sucrose (Table 2, entry 2) bearing only 5 mmol/g aldehyde (Table 1, entry 2), and the result showed substantially reduced overall mechanical strength (Table 2, entry 3). This confirmed our conclusion, and this trend is not surprising since aldehyde groups were responsible for the crosslinking, so the higher the aldehyde content, the higher the mechanical strength.

On the other hand, the oxidized cyclic oligosaccharides (Table 2, entry 6,7 and 8) all showed lower mechanic strength than oxidized sucrose did, although their aldehyde content was all higher than sucrose. This difference may be caused by the steric hindrance. The hydrodynamic radii of α-, β-and γ-cyclodextrin are 6.7 Å, 7.7 Å and 8.5 Å [25] respectively, which are all larger than that of sucrose (4.5 Å [26]). Since the structure of the oxidized oligosaccharides largely resembles that of their precusor, it is reasonable to conclude that oxidized cyclodextrins are considerably larger than oxidized sucrose. As a result, the former had a slower diffusion rate than the latter and was unable to accumulate enough concentration in the sea cucumbers to perform the same stabilization. This argument is further supported by the fact that the treatment of dialdehyde starch, a polymeric multialdehyde, to sea cucumbers only marginally improved their heat stability. Furthermore, in the case of oxidized stachyose (Table 2, entry 5), the molecule was both under-oxidized and sterically hindered (hydrodynamic radius = 6.4 Å [26]); thus, it also provided minor stability enhancement which is in accordance with the aforementioned explanation.

**Figure 2:**
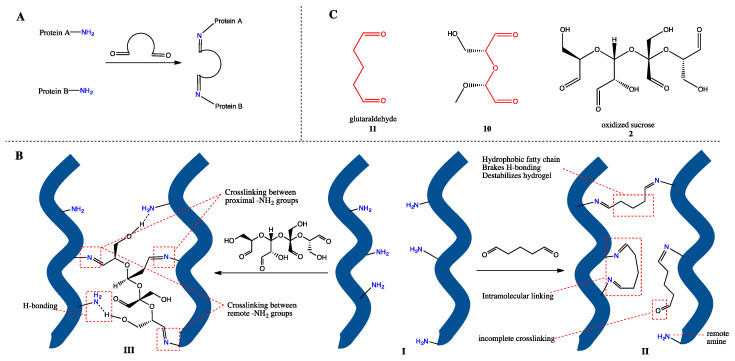
Mechanism of crosslinking. (**A**) Concept of the crosslinking process. (**B**) Crosslinking protein chains with glutaraldehyde (I→II) and oxidized sucrose (I→III). (**C**) Compound 10 resembles partial features of both glutaraldehyde (11) and oxidized sucrose (2).

Glutaraldehyde is probably the most popular small-molecule crosslinker and is often used for mechanical enhancement of amine-group-bearing macromolecule-based materials, such as chitosan [27] and protein [28]. Thus, a comparison with glutaraldehyde (Table 2, entry 10) would provide a strong reference to the performance of oxidized oligosaccharides. To our delight, both oxidized α-, β-and γ-cyclodextrin were comparative with glutaraldehyde in stabilizing rehydrated sea cucumbers, and fully oxidized sucrose even outperformed glutaraldehyde. This result is somewhat inconsistent with the previous conclusion that higher aldehyde content and smaller molecular size is preferred for better stabilization since glutaraldehyde has the highest aldehyde content (20.0 mmol/g) and is the smallest tested molecule in this research. We proposed two reasons to explain this paradox:

(1) Glutaraldehyde is less hydrophilic than oxidized sucrose. Protein hydrogel is a 3D network mainly stabilized by water-bridged hydrogen bondings. The fatty chain of glutaraldehyde is hydrophobic, so the introduction of this moiety to hydrogel is expected to break the proximal hydrogen bonding network and thus compromise the overall stabilizing effect. On the other hand, oxidized sucrose provides multiple hydrophilic groups that could serve as hydrogen bonding donors or acceptors and is thereby considered benign to the local hydrogen bonding network.
(2) Glutaraldehyde has a shorter chain and fewer aldehydes per molecule than oxidized oligosaccharide does. The mechanism of crosslinking is imination between a crosslinker and at least two amines from different peptide chains (Figure 2A), which covalently connects the chains and thus stabilizes the hydrogel. This process is reasonably easy in solution, where molecules have high degree of freedom, yet is considerably harder in a gel system, because in the latter, the functional groups are “confined” to a relatively small space by various inter- and intra-molecular interactions and, as a result, cannot react as “freely” as they would in solution. For glutaraldehyde, whose reactivity in sea cucumber body wall is restricted by its short chain length, when no heteromolecular amine exists in the proximal, half-reacted glutaraldehyde may either form an intramolecular link with a proximal amine in the same protein or, in rarer cases, remain with the other end unreacted (Figure 2B, I→II), which both result in incomplete crosslinking. However, for oxidized sucrose, multiple aldehyde groups are located at different positions along a longer chain, which provided the accessibility and flexibility to react with both proximal and remote amine groups in the gel network. In addition, even if an intramolecular link should occur, since each oxidized sucrose bears four aldehyde groups, the remaining ones still may react intermolecularly, providing higher possibility for crosslinking (Figure 2B, I→III).

In order to test these two assumptions, we designed and synthesized compound 10 from methyl glucoside, which resembles the chain length of glutaraldehyde (Figure 2C, red) but provides extra hydroxide groups and oxygen atoms that were expected to behave as hydrogen bonding donors and acceptors. Thus, if explanation (1) holds, compound 10 crosslinked sea cucumbers would be more heat-stable than glutaraldehyde crosslinked ones due to more hydrogen bonding possibilities. On the other hand, the molecule is inferior to oxidized sucrose in terms of chain length and aldehyde number. If assumption (2) holds, compound 10 crosslinked sea cucumbers would be less heat-stable than oxidized sucrose crosslinked ones. The texture profile of compound 10 crosslinked HTT sea cucumber is listed in Table 2 (entry 11), which, in general, is between glutaraldehyde and oxidized oligosaccharide (Table 2, entry 11 and 3, respectively). This result was well in accordance with our prediction and thus proved that assumption (1) and (2) are both correct. Furthermore, this set of experiments has also suggested that the mechanism of stabilization from oxidized oligosaccharide involves not only covalent crosslinking but also hydrogen bonding formation.

### 2.3. Degree of Imination and Total Soluble Material

The major source of covalent stabilization is the formation of imines between aldehydes and amines. Thus, a comparison of residue amines would provide useful information to estimate the efficiency of the reactions. However, imines are susceptive to nucleophilic attack from the electron lone pairs on -NH_2_ or -OH to form 2° amines that are also responsive to many amine detecting methods (e.g., ninhydrin method). In order to avoid this uncertainty, the o-phthalaldehyde method is a good choice [29], which selectively responds to 1° amines over 2° amines under the test condition.

The three oxidized oligosaccharides that provided higher stabilization (according to the texture profile analysis) to rehydrated sea cucumbers were chosen for the test, including oxidized sucrose, raffinose and β-cyclodextrin, and their degree of imination was calculated with reference to the control group. As listed in Table 3, all crosslinked samples showed the minor degree of imination (1–3%), yet interestingly, these numbers were all proven statistically non-zero according to one-sided (H_0_: μ = 0, H_1_: μ > 0) Welch’s *t*-test at *p* < 0.05 level. This result indicated that these small molecules are highly efficient in stabilizing sea cucumber proteins, and a low degree of imination is sufficient for a satisfactory enhancement to the protein hydrogel.

A very important feature of deterioration after high-temperature treatment is the increase of total soluble material. We then extracted and weighed the total soluble material of uncrosslinked HTT sea cucumbers and collected 25.34% total soluble material. In contrast, oxidized sucrose, oxidized β-cyclodextrin, and oxidized raffinose crosslinked group only yielded 7.78%, 10.44% and 13.60% total soluble material, respectively (Table 3). This set of data, when analyzed together with Table 2, showed that better overall mechanical performance corresponded to lower total soluble material (overall mechanical performance: oxidized sucrose > oxidized β-cyclodextrin > oxidized raffinose according to Table 2). This relationship is not surprising because lower total soluble material is equivalent to higher structural integrity of the protein walls, and as a result, exhibited stronger mechanic profile.

Furthermore, these results also caused us to reconsider the mechanism of deterioration. Our group previously suggested that high-temperature treatment caused thermal breakage of chemical bonds in collagen fiber, which in turn induced disorder to gel structure that eventually destabilized the body wall, and suspected that both hydrogen bonds and peptide bonds were broken during the process [21]. However, the present work inferred that the breakage of peptide bond is probably not responsible for the deterioration. Peptide bond, in essence, is an amide bond that is more chemically stable against hydrolysis than imines at near-neutral pH. If amide bonds were to have broken during heating, the oxidized oligosaccharide based imine crosslinking would have been severely hydrolyzed and would have provided hardly any stabilization. This assumption is clearly contrary to the texture profile analysis results obtained in this study. Therefore, the breakage of amide bonds (peptide bonds) is excluded from the major reason for the deterioration. Accordingly, we now suggest that the mechanism of deterioration is more likely a process of relocation of hydrogen bonding linkage accompanied with water redistribution, which is somewhat similar to the pasting of starch.

### 2.4. Appearance and SEM

**Figure 3:**
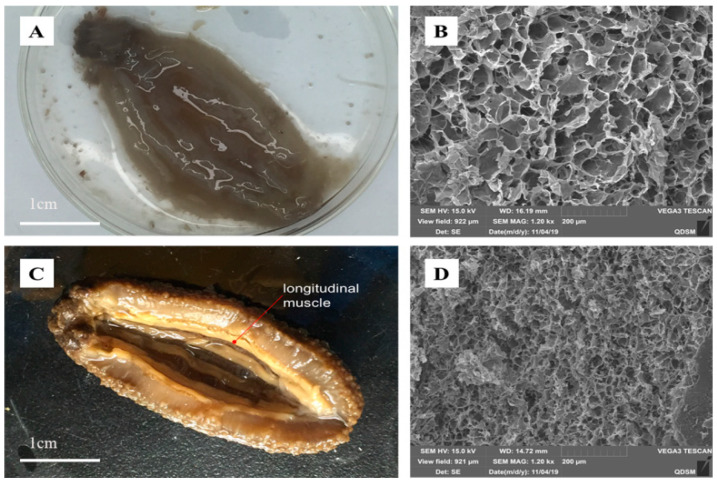
Appearance and SEM images of the uncrosslinked and oxidized sucrose crosslinked high-temperature-treated (HTT) rehydrated sea cucumbers. (**A**) Appearance of the uncrosslinked. (**B**) SEM image of the uncrosslinked. (**C**) The appearance of the crosslinked. (**D**) SEM image of the crosslinked.

Figure 3A shows the typical appearance of a deteriorated HTT rehydrated sea cucumber, which has an ambiguous outline as well as soft-looking texture, and difficult to observe structural details. In contrast, oxidized sucrose crosslinked rehydrated sea cucumber (Figure 3C) showed a clear and distinct outline and good sensory texture. In addition, the structural details of sea cucumber (such as the fibrous body wall and the longitudinal muscle) could easily be spotted. Obviously, the above results are in accordance with the texture profile data. The rehydrated sea cucumber crosslinked with oxidized sucrose was superior to the control group in terms of hardness, resilience, chewiness and springiness. The SEM images of the two samples were both well in accordance with their appearance. Under SEM, the uncrosslinked (Figure 3B) presented a highly porous structure with fibers loosely distributed in space. The formation of this structure was probably due to the solvation of body wall proteins and the disintegration of fibers at high temperature. On the other hand, the SEM image (Figure 3D) of the crosslinked samples showed that the pores in the body wall of sea cucumber were small and dense, indicating that the internal structure was complete and fibers were tightly bound. This phenomenon fully confirmed that the small molecule oxidized oligosaccharides could freely diffuse into sea cucumber body walls, and covalently crosslinked with amine groups from different peptide chains, which stabilized the 3D network structure of sea cucumber at high temperature. In conclusion, these images have clearly shown that the oxidized oligosaccharide successfully protected the rehydrated sea cucumber from heat impact and maintained its appearance.

## 3. Materials and Methods

### 3.1. Materials

Salted sea cucumbers (Apostichopus japonicus) were purchased from Nanshan fish market (Qingdao, China), and stored in laboratory −18 °C refrigerator.

All sugars were purchased from Shanghai Yuanye Biotechnology Co., Ltd. (Shanghai, China); Sodium periodate, methanol, ethyl acetate, hydroxylamine hydrochloride, sodium hydroxide, acetic acid, glutaraldehyde, ethanol, o-phthaldialdehyde, SDS, β-mercaptoethanol, Sodium tetraborate, L-lysine etc. were all purchased from Sinopharm Group Chemical Reagent Co., Ltd. (Shanghai, China); Dialdehyde starch was purchased from Taian Jinshan Modified Starch Co., Ltd. (Shandong, China).

All reagents were of analytical grade or the best grade available.

### 3.2. Synthesis of Oxidized Oligosaccharides

#### 3.2.1. Synthesis of Normally Oxidized Oligosaccharides

In the present study, normally oxidized sucrose, raffinose, stachyose, α-, β-and γ-cyclodextrin were prepared using this method. The amount of NaIO_4_ used for oxidation was calculated according to the total number of monosaccharide units present in one oligosaccharide molecule, and for each monosaccharide unit, 1.1 equivalence (eq.) of NaIO_4_ was used [30]. Oligosaccharide was dissolved in H_2_O to 0.2 M and cooled to 0 °C in a round bottom flask covered with aluminum foil. Then NaIO_4_ was added portionwise to the solution under vigorous stir. The resulting solution was stirred in darkness for 2 h at 0 °C and then allow to warm to room temperature and stirred for another 18 h in darkness. Then the solvent was removed in vacuo, and the inorganic salts were removed by a short column to yield the normally oxidized oligosaccharides. ^1^H nuclear magnetic resonance (^1^H NMR) was used to characterize the oxidized oligosaccharides. The sample was dried under reduced pressure and redissolved in D_2_O. A 400 MHz NMR spectrometer (Ascend 400, Bruker, Switzerland). was used to take the spectra.

#### 3.2.2. Synthesis of Partially Oxidized Sucrose

To a stirred solution of NaIO_4_ (1 eq., 0.2 M) was added sucrose (1.0 eq.) at 0 °C and the solution was stirred for 28 h in darkness. After completion of the oxidation, the solvent was evaporated in vacuo and the inorganic salts were removed by a silica gel short column (3:7 Methanol-ethyl acetate) to yield the partially oxidized sucrose.

#### 3.2.3. Synthesis of Fully Oxidized Methylglucoside

To a stirred solution of NaIO_4_ (3.0 eq., 0.2 M) in H_2_O was added methyl α-D-glucopyranoside (1.0 eq.) at 0 °C. The solution was further stirred for 24 h in darkness. After the completion of oxidation (detected by TLC), the solvent was evaporated and inorganic salts were removed by a short silica gel column (3:7 Methanol-ethyl acetate) to yield the fully oxidized methylglucoside.

### 3.3. Rehydration of Dry Sea Cucumber

The salted sea cucumbers were desalted for 24 h in 5 × volume of water (refreshed once per 12 h) at 4 °C. After desalination, intestines and teeth were removed and the sea cucumbers were boiled for 1.5 h. Then the sea cucumbers were soaked in 5 × volume of water at 4 °C for 48 h for full rehydration.

### 3.4. Crosslinking of Rehydrated Sea Cucumber and High-Temperature Treatment

Rehydrated sea cucumbers were soaked in aqueous solutions of oxidized oligosaccharides (0.5% *w*/*v*) in Erlenmeyer flasks. The flasks were then heated in a water bath at 80 °C for 20 min, then cooled to room temperature, and then sealed and heated at 121 °C for 10 min in a sterilizer and finally allowed to naturally cool to room temperature.

### 3.5. Texture Profile Analysis (TPA)

TMS-TOUCH texture analyzer (Food Technology Corporation, Sterling, VA, USA) with a cylindrical probe of 75 mm in diameter was used for TPA measurements. Each sample was compressed twice to 65% of its original height, and the time interval between two compression cycles was 5 s. The force–time curve was obtained with a crosshead speed of 30 mm/min and a starting force of 1 N. Five parallel experiments were carried out on each sample.

TPA parameters obtained from the force–time curve were hardness, springiness, resilience and chewiness [31].

### 3.6. Aldehyde Content

The determination of aldehyde content was conducted according to the method reported by Maute et al. [24] with minor modifications. Briefly, hydroxylamine hydrochloride (8.69 g, 0.125 mol) was dried in a thermostatic air drying oven at 110 °C to constant weight, dissolved in 75 mL pure water, dropped with methyl orange aqueous solution (3 mL, 0.05%) and fixed volume to 500 mL. In addition, 0.1 g of oxidized oligosaccharide was dissolved in 25 mL of the abovementioned solution and stirred for 2 h. Then the solution was titrated with 0.100 mol/L NaOH. For each oxidized oligosaccharide, three parallel titrations were conducted.

The aldehyde concentration ((CHO), mmol/g) was calculated using the following formula:

(1) ΔV×0.001×nNaOH=nCHO

(2) $$[CHO]=1000nCHO/W_{o}$$

where Δ*V* denotes the volume of NaOH consumed, in mL; nNaOH denotes the molar concentration of NaOH, in mol/L; nCHO denotes the molar number of aldehyde group on oxidized oligosaccharide; W_o_ denotes the mass of oxidized oligosaccharide, in g.

### 3.7. Total Soluble Content

Freeze-dried sea cucumber powder (0.500 g) was rehydrated in 40 mL ultra-pure water, and heated in a water bath at 37 °C for 20 min, and then extracted with ultrasonic assist for 2 h. The mixture was then centrifuged at 10,000 r/min for 15 min, and the supernatant was collected and freeze-dried with freeze dryer (FD-1D-50, Beijing boyiakang Experimental Instrument Co., Ltd., Beijing, China) and the residue was weighed. All experiments were run in triplicate. The total soluble content was calculated according to the following formula:

(3) $$\mathrm{The}\;\mathrm{total}\;\mathrm{soluble}\;\mathrm{content}\;(\%)=W_{f}{/W}_{0}\times 100\%$$

where, W_0_ denotes the total mass of the sea cucumber powder sample before extraction, in g; W_f_ denotes mass of soluble material, in g.

### 3.8. Degree of Amination

The degree of amination was determined according to the method reported by Hare et al. [29] with minor modifications: Freeze-dried sea cucumber samples were pulverized, and a sample of 10 mg powder was dissolved in acetic acid solution (0.5 M, 200 µL) and ultrasonic-extracted for 40 min. Then the solution was treated with NaOH (0.5 M) to neutral pH and centrifuged at 5000 r/min for 15 min by a refrigerated centrifuge (TLG-16M, Hunan Xiangyi Centrifuge Instrument Co., Ltd., Hunan, China). The supernatant (100 µL) was incubated in 3 mL OPA reagent at ambient temperature in darkness for 5 min. For each oxidized oligosaccharide crosslinked sea cucumber, three parallel titrations were conducted.

Preparation of o-phthaldialdehyde (OPA) reagent: OPA (80 mg) was dissolved in ethanol (2 mL, 95% *w*/*v*). To the solution was then added sodium tetraborate buffer (50 mL, 0.1M, pH 9.5), SDS (5 mL, 20% *w*/*v*) and β-mercaptoethanol (0.2 mL). The resulting solution was then fixed volume to 100 mL with deionized water. Absorbance was recorded by a spectrophotometer (UV-2102PC, Unico, Shanghai, China) at the wavelength of 340 nm. Deionized water was used as the blank. The content of free amino group was then calculated according to the calibration curve, using Lysine (0–5 mmol/L) as standard.

### 3.9. Ultrastructural Observation of Samples

The tested rehydrated sea cucumbers were cut into 1.0 cm × 1.0 cm × 0.5 cm tissue blocks, which were frozen and fixed at −80 °C for 2 h and then freeze-dried. After sputtering the samples with ion sputtering apparatus, the samples were observed with scanning electron microscope (SEM) (S-4800 Hitachi, JEOL, Tokyo, Japan). The magnification was 1200 times [32].

### 3.10. Statistical Analysis

The experimental data were analyzed by SPSS 22 software (SPSS Inc., Chicago, IL, USA). *p* < 0.05 was considered significant.The data were expressed as means ± SD.

## 4. Conclusions and Future Work

This pilot study has confirmed that oxidized oligosaccharides could efficiently diffuse into the body wall of rehydrated sea cucumbers and crosslink the collagen system. The crosslinked sea cucumbers were more stable against heat-promoted deterioration than the control group according to the mechanical stability assay, and the oxidized sucrose crosslinked group exhibited the best overall performance. Both covalent bond and hydrogen bonding were found responsible for the improvement in heat stability; aldehyde content was found to positively correlate with the stabilizing effect, yet molecular size was negatively correlated. In addition, the degree of imination assay showed that for a satisfactory stabilization, only 1–3% amine groups were required for imination, indicating the crosslinkers were highly efficient in stabilizing the hydrocolloid system. Furthermore, the crosslinking successfully reduced the weight loss and maintained the appearance of sea cucumbers during the high-temperature treatment. The results of this study have suggested that oxidized oligosaccharide, especially oxidized sucrose, could be used as a small-molecule stabilizing agent, which could diffuse into protein hydrocolloids without compromising its appearance and structural integrity and hence could have great potential in food industry.

On the other hand, before oxidized oligosaccharide could be used in real-world production of foods, more biological and food safety information must be gathered. Therefore, a substantial amount of work is required to elucidate their toxicology and metabolism profiles.

## 5. Patents

The authors have filed a patent application covering the process: Chinese patent application no. 201911366769.4.

## Figures and Tables

**Table 1:** Actual and theoretical aldehyde content of the oxidized saccharides.

Entry	Oxidized Oligosaccharide	Actualaldehyde Content (mmol/g)	Theoretical Aldehyde Content (mmol/g)
1	Sucrose ^a^	5.04 ± 0.162	5.88
2	Sucrose ^b^	9.14 ± 0.049	12.99
3	raffinose	6.83 ± 0.029	12.05
4	stachyose	7.73 ± 0.123	12.16
5	α-cyclodextrin	10.08 ± 0.038	12.50
6	β-cyclodextrin	9.54 ± 0.116	12.50
7	γ-cyclodextrin	9.94 ± 0.166	12.50
8	methyl glucoside	12.31 ± 0.12	12.34
9	Dialdehyde starch	4.7 ± 0.17	12.50

^a^ Sucrose, partially oxidized: each mole of sucrose was oxidized with 1.1 mole of NaIO_4_. ^b^ Sucrose, normally oxidized: each mole of sucrose was oxidized with 2.2 mole of NaIO_4_.

**Table 2:** Texture profile of HTT sea cucumbers.

Entry	Crosslinker ^1^	Hardness (N)	Chewiness (mJ)	Resilience (N)	Springiness (mm)
1	control	20.28 ± 4.74 ^a^	15.56 ± 7.56 ^a^	10.76 ± 3.36 ^a^	1.39 ± 0.35 ^a^
2	sucrose ^2^	36.38 ± 4.12 ^bcd^	60.21 ± 9.9 ^bcd^	23.55 ± 0.79 ^bcde^	2.55 ± 0.35 ^cd^
3	sucrose ^3^	61.2 ± 5.28 ^f^	138.67 ± 32.15 ^e^	48 ± 5.57 ^f^	2.89 ± 0.56 ^df^
4	raffinose	45.3 ± 3.71 ^def^	94.34 ± 9.17 ^de^	31.15 ± 2.71 ^def^	3.04 ± 0.31 ^f^
5	stachyose	34.2 ± 11.28 ^bc^	52.04 ± 31.59 ^abc^	23.75 ± 9.31 ^bcd^	2.07 ± 0.42 ^b^
6	α-cyclodextrin	33 ± 5.58 ^bc^	41.17 ± 7.45 ^ab^	21.4 ± 4.3 ^bc^	1.93 ± 0.08 ^b^
7	β-cyclodextrin	44.35 ± 6.81 ^de^	80.24 ± 21.67 ^cd^	31.3 ± 6.53 ^cde^	2.54 ± 0.24 ^cd^
8	γ-cyclodextrin	43.5 ± 8.46 ^cde^	73.81 ± 14.55 ^bcd^	31.68 ± 7.16 ^cde^	2.35 ± 0.13 ^bc^
9	dialdehyde starch	29.88 ± 3.79 ^ab^	35.93 ± 7.6 ^ab^	18.9 ± 2.46 ^ab^	1.89 ± 0.21 ^b^
10	glutaraldehyde	44.1 ± 13.84 ^cde^	74.82 ± 34.94 ^bcd^	31.53 ± 12.5 ^bcde^	2.29 ± 0.33 ^bc^
11	methyl glucoside	58.25 ± 1.2 ^ef^	115.72 ± 28.97 ^de^	38.55 ± 7.85 ^ef^	2.99 ± 0.14 ^df^

Different superscript letters (a-f) in the same column indicate significant differences (*p* < 0.05). ^1^ All sugars listed in this column refer to the ones oxidized with NaIO_4_; ^2^ sucrose, partially oxidized: each mole of sucrose was oxidized with 1.1 mole of NaIO_4_; ^3^ sucrose, normally oxidized: each mole of sucrose was oxidized with 2.2 mole of NaIO_4_.

**Table 3:** Degree of imination measurement results of HTT sea cucumbers. The results are presented as Average ± SD of triplicate experiments.

Entry	Crosslinker	Degree of Imination (%)	Total Soluble Material (%)
1	None (control)	-	25.34
2	oxidized sucrose	2.796 ± 0.0024 *	7.78
3	oxidized raffinose	2.023 ± 0.0005 *	13.60
4	Oxidized β-cyclodextrin	1.011 ± 0.0025 *	10.44

* Result significantly non-zero at *p* < 0.05 level. “-” indicates no imination.

## Abbreviations

HTT	High-temperature treated
TLC	Thin Layer chromatography
TPA	Texture profile analysis
OPA	O-phthaldialdehyde
SDS	Sodium dodecyl sulfate
SEM	Scanning electron microscope
NMR	Nuclear magnetic resonance
